# Reviewing the complex link between puerperium and psychosis: a case report

**DOI:** 10.1192/j.eurpsy.2022.2221

**Published:** 2022-09-01

**Authors:** E. Herrero Pellón, P. Albarracin, M. Huete Naval, R. Galerón, A. García Recio

**Affiliations:** Hospital Clínico San Carlos, Institute Of Psychiatry And Mental Health, Madrid, Spain

**Keywords:** Psychosis, puerperium, woman, Pregnancy

## Abstract

**Introduction:**

We present the case of a 23-year old woman with a history of two hospitalizations in the psychiatric ward of our hospital in the last 8 months. Prior to this age our patient had not required assistance from mental health professionals. The wide variety of symptoms shown by the patient included auditive hallucinations and persecution delusions that led to behavioral alteration and depressive symptoms.

**Objectives:**

To present a case report of a puerperal psychosis and to review the different kind of psyquiatric disorders that may arise in the puerperium.

**Methods:**

Literature review of scientific papers over the last years and classic textbooks on the issue. We included references in English and Spanish languages.

**Results:**

During pregnancy and the puerperium there are biochemical, hormonal, psychological and social changes that cause a vulnerability in women for the appearance of mental disorders. The differential diagnosis of puerperal psychoses must first be made with organic diseases. Once this has been discarded, several studies indicate that there is a high probability that after the onset of puerperal psychosis a cyclical mood disorder is found.

**Conclusions:**

- One of the main characteristics of puerperal psychoses is the great variety of its symptomatic manifestations. They can present characteristics of both mood disorders and schizophreniform disorders. - Deep confusion and delusions are often the most prominent symptoms of psychosis in the puerperal period.

**Disclosure:**

No significant relationships.

